# The influence of school climate on teachers’ job satisfaction: The mediating role of teachers’ self-efficacy

**DOI:** 10.1371/journal.pone.0287555

**Published:** 2023-10-05

**Authors:** Jie Fang, Zhanyong Qi

**Affiliations:** Faculty of Education, Shaanxi Normal University, Xi’an, China; Sichuan Agricultural University, CHINA

## Abstract

Improving the job satisfaction of vocational education teachers (VET) is necessary for maintaining the stability of the vocational education teaching force and is an essential support for enhancing the professional attractiveness and ensuring the quality of VET. Unlike previous studies, firstly, in terms of groups, the particular group of teachers in this study focuses on VET, which in reality is often overlooked compared to other groups of teachers due to the low level of recognition of vocational education (VE) in China. Secondly, this phenomenon is more prominent and less researched regionally in economically underdeveloped areas due to the uneven and disparate economic development between the East and West of China. In terms of sample size, previous studies have lacked large samples, making it difficult to obtain convincing and realistic results. Finally, most previous studies considered teachers’ job satisfaction (TJS) in terms of external or internal factors alone. However, this study considers a combination of the external factor of the school climate (SC) in which VET works and the internal factor of teachers’ self-efficacy (TSE) to explore the relationship with TJS and its impact. Based on data from a survey of 1035 teachers in China, a structural equation model estimation method was used to explore and analyze the impact of SC and TSE on their TJS and the specific role of the relationship. The research found that VET-perceived SC is positively predicting TJS; VET-perceived SC is positively predicting TSE; TSE is positively predicting TJS; TSE significantly mediates the relationship between SC and TJS. This study provides a theoretical perspective that integrates the external organizational influences of SC and introduces the internal psychological factor of TSE as a mediating variable to understand TJS. In practice, it helps raise the importance of VET and provides empirical data and intellectual support to the relevant government departments in improving TJS.

## Introduction

A high-quality vocational education (VE) system is the hallmark of a new stage of development in VE. As a “booster” for the reform and development of VE, teachers play an essential role in improving the quality of talent training, deepening the integration of industry and education for upgrading, and enhancing the ability to serve national strategic development. Developed countries have permanently attached great importance to the development of VE and paid particular attention to the training of vocational education teachers (VET), starting as early as the 17th century with the introduction of many bills and policies aimed at strengthening teachers’ job satisfaction (TJS) in the form of national legislation and policy documents. Education is contextual and territorial. Most studies have been conducted to explore the issue of TJS in a Western context, but there needs to be more studies in non-Western contexts. In China, due to the influence of Confucianism, represented by Confucius and Mencius, technical skills are often despised and belittled. As time passes and overlaps, such ideas are retained across time and space. As a result, VET are often overlooked compared to other teacher groups, particularly in economically backward areas of central and western China. The great peoples’ educator Ushinsky once said, “Teachers are the bridge between all the beautiful and noble things in human history and the new generation.” Teachers are the foundation and the source of education. VET are the primary bearers of training technically skilled talents, which is extremely important for improving the level and quality of vocational education. Improving the TJS of VET is the key to running vocational education well.

Most domestic and international studies have focused on the influence of a single factor on TJS. However, there is a lack of a comprehensive examination of the influence of the external factor of teachers’ perceived school climate (SC) and the internal factor of teachers’ self-efficacy (TSE) on TJS and how the interaction between internal and external factors works. The development of high-quality VE cannot be achieved without the critical resource of teachers. Therefore, it is imperative to address the TJS problems faced by VET, especially in economically underdeveloped areas. TJS is a complex issue that involves both internal and external factors. What are the relationships between them? How can solutions be developed? This has not yet received the attention and discussion it deserves.

Mcnichols et al. [1] argue that job satisfaction represents the overall attitude of employees toward their jobs, i.e., their intrinsic feelings and satisfaction with the job itself and the events, people, and environment related to the job. It is an essential variable in organizational behavior and human resource management and has received attention from researchers.

This is no exception in the field of education. According to the research of Lopes et al. [2], Toropova et al. [3], and Buonomo et al. [4],TJS has been a significant issue of academic research over the last decade, and this trend has been more evident in all regions of the world. Skaalvik et al. [5] view TJS as teachers’ emotional response to their educational and teaching work or school work environment. As an essential indicator of teachers’ professionalism, it directly affects teachers’ motivation and professional development. In today’s world, teachers are a vital factor that cannot be ignored for schools to reach specific educational quality goals. Teachers need to be happy and appreciated in their work, and meeting these expectations makes them feel satisfied with their school and contribute to it positively. When teachers are confident in their work’s economic benefits and their colleagues’ interpersonal relationships, their morale is bound to increase [6].

Whether teachers can produce the quality of human resources required by society at the behest of political interests such as the government, or they can work in partnership with the school principal to promote the quality of education within the school, and or their performance earns the respect of students and parents, are all closely related to the SC that teachers perceived. The management style of the principal, the level of teacher involvement in school affairs, job satisfaction, self-efficacy, and the harmonious relationships between colleagues, teachers, and students are all highly dependent on the SC as perceived by teachers. As Perret [7] argues, SC refers to the “esprit de corps”. Freiberg et al. [8] define SC as “the heart and soul of a school, the essence of a school that leads students, teachers, and administrators to love the school and look forward to every day of school life”. It is also “the atmosphere, culture, resources, and social networks of a school” [9].

Teachers’ perceived SC is their experience of school life and reflects the norms, goals, values, relationships, teaching and learning practices, and organizational structure of a school [10]. Also is the heart and soul of the school as a reflection of the schools’ team spirit [11]. Banks et al. [12] and Vedder et al. [13] believe that teachers’ perceptions and attitudes significantly impact the SC of schools and students’ perspectives. These shared perceptions and attitudes about the schools’ SC allow individuals to understand ambiguous and conflicting organizational cues. SC improvement is a proven school improvement strategy for building safer, more supportive, and more civilized schools. Research has demonstrated that in a well-integrated school environment, teachers are more likely to take the initiative to develop and demonstrate their personal competencies and to strive for support to achieve their goals. Teachers who are not supported in their teaching feel unmotivated [14,15].

Educational researchers, practitioners, and policymakers generally agree that TSE is a fundamental characteristic of teachers and is closely related to their teaching practices and the quality of their teaching [16]. It has been suggested [17] that TSE significantly impacts their goal setting, commitment to their work, and ability to continue to teach when they encounter difficulties. TSE is highly valued internationally in individual countries and large-scale educational assessments. Enhancing the TSE of VET is a critical issue in the current development of the teaching force in vocational colleges.

However, do the previously mentioned teachers’ perceived SC directly affect TJS and TSE? Are there differences in TJS and TSE across teachers’ perceived SC? What is the relationship between teachers’ perceived SC, TSE, and TJS? To date, little research has focused on this question. Through a survey of 1,035 VET in western China, this study attempts to examine the internal and external factors influencing TJS of VET from a multivariate perspective through empirical analysis to explore the influence of SC, an external factor, on TJS and what role TSE, an internal factor, plays in this so as to provide theoretical references and practical insights for school management and teacher development.

## Literature review

### School climate

The study of organizational climate first originated from Litwin’s [18] field theory, which was used to study the influence of the environment on individuals. He believed that the prerequisite for understanding individual behavior is to understand the specific environment in which their behavior is generated, and he defined the organizational climate as the similar feelings generated by executive members who perceive the corporate environment directly or indirectly. Some scholars [19] consider the sense of organizational climate as the perception of collectivism, stability, etc., by the organization’s members. In the 1960s, Halpin et al. [20] introduced organizational climate to the school sector, calling it School Organizational Climate (SOC), which is to the organization what personality is to the individual and considers SOC to be the organizational personality of the school.

School is also an organization. Some scholars [21] consider SC to be the quality and frequency of interpersonal interactions among all members of the school that affect children’s cognitive, social, and psychological development. As an environmental variable, SC originated in the study of effective schools. Cohen et al. [22] and Wilson [23] believe that school is the most critical environment for adolescents to grow up in, other than the family. It is not only a place for adolescents to learn and develop cognitively but also an essential background to form positive social relationships and develop emotionally and behaviorally. The concept of SC was first introduced by Perry [24] in The Management of a City School, where he stated that “all school personnel should value and participate in the building of a school climate and cultivate a sunny school atmosphere, not limit to providing a place for students to learn”.

Researchers [25,26] have, in turn, defined and measured it in various ways for their research purpose. Combining psychological and organizational perspectives, Cohen et al. [27] argue that SC is a reflection of multiple aspects of school regulations, goals, values, interpersonal relationships, teaching and learning practices, and organizational structures based on school members’ experiences of school life, and produces relatively persistent and stable environmental features of teachers’ behavior that reflect the quality and character of school life. Hoy et al. [28,29] build on their predecessors by further defining SC in terms of an organizational health model as relatively stable characteristics that affect its members and that are used to distinguish different schools. Dubbeld et al. [30] argue that teachers’ perceived SC is their experience of school life and reflects the norms, goals, values, relationships, teaching and learning practices, and organizational structure of a school.

In the context of the aims of this study, we adopt Hoy et al.’s [31] definition of SC from the perspective of teachers’ perceptions as a set of intrinsic characteristics that distinguish a school from other schools and influence the behavior of each school member. Specifically, SC is a relatively enduring characteristic of the school environment experienced by its members and influences their behavior based on their collective behavioral perceptions.

Numerous studies have shown that SC has a profound impact on the physical and mental health of teachers. SC reflects all aspects of a student’s school experience, including the quality of instruction, school community relations, school organization, and the disciplinary and structural characteristics of the school environment, determine the quality of student interactions with teachers, parents, and school personnel, and reflects the norms, values, and goals of the overall educational status and social mission of the school [32]. The finding of Gregory et al. [33] demonstrates that transparent school systems and strong school support can reduce the threat to teachers. Singh et al. [34] suggest that teachers are more committed to their work if they feel supported by their principals and colleagues. Grayson et al. [35] confirm the impact of SC on faculty members’ emotional exhaustion and depersonalization.

In cross-national studies of SC, scholars have tended to choose the path of cultural differences to cut through to understanding. The finding [36] argue that the effects of power distance are particularly pronounced in schooling, where in cultures with high power distance (East Asian countries), schools are teacher-centered and disciplined, and teachers are seen as guardians of knowledge; in cultures with low power distance (Western countries), schooling is learner-centered, and teachers treat students more equally. Other scholars [37] have suggested that East Asian countries have a more positive learning environment due to their strict school discipline and culture of respect for teachers.

SC contains different perceptual subjects, such as students, teachers, and administrators. Mitchell et al. [38] found that teachers and students perceive SC differently. The subject of this study is teachers, so the perceptual subject of SC should also focus on teachers. Specifically, SC is a relatively persistent characteristic of the school environment experienced by teachers and influences their behavior based on their collective perceptions of behavior. Research [39,40] has categorized teachers’ perceptions of SC into three dimensions: shared decision-making, collegiality, and student-teacher relationships. There is no consensus on the meaning of SC, and no unified measurement criteria have been given. However, the core factors are the institutional and interpersonal aspects of the school. It involves both institutional climates at the organizational level, such as management decision-making style, and within the organization, such as collegiality and student-teacher relationships. Distributed leadership is positively and indirectly correlated with TJS and TSE. [41] Decision-making sharing is about increasing teachers’ participation in school decision-making and changing from “top-down” authoritative leadership to “bottom-up” caring and democratic leadership. John [42] suggested that principal concern for leadership was positively associated with an open SC. Woolfolk et al. [43] found that the collegial relationship was one of mutual respect, mutual trust, mutual concern, and frank exchange of ideas among teachers.

### Teachers’ job satisfaction

Job satisfaction, also called someone who is satisfied with the job, was first introduced in 1935 by scholar Hoppock [44], who believed that job satisfaction is the psychological and physical satisfaction of employees with environmental factors, that is, the subjective response of workers to the work situation.

Different scholars have different perceptions of job satisfaction. Locke [45] considered job satisfaction as a pleasant emotional state that stems from a positive evaluation of the realization of one’s work value. It represents employees’ overall attitude toward their jobs and the intrinsic feeling of satisfaction and satisfaction with the job itself and the events, people, and environment associated with the job [46]. It has been argued by Hocl et al. [47] that job satisfaction refers to the pleasant or positive emotional state brought about by one’s job and work experience, as well as an indicator for individuals to assess their job achievements to prove the value of their work. Worrell et al. [48] also considered it a reflection of peoples’ work emotions in the work process.

Teaching is a profession with unique attributes. Landy [49] viewed TJS as a psychological concept that refers to teachers’ general emotional feelings and perceptions about their jobs and careers, as well as their working conditions and situations. It is a public view and sense of teachers’ occupation [50]. Or refers to teachers’ emotional reactions to their educational and teaching work or school environment [51].

Research [52] showed that TJS affects not only their job status and professional development but also the effectiveness of education and teaching and the quality of talent development. It also affects their teaching effectiveness and ultimately affects students’ learning and healthy development [53].

High TJS produces many positive results. It has been established that TJS is significantly and positively related to organizational personal behavior [54], affecting teachers’ work ethic [55] and reducing faculty motivation to leave [56], etc.

Regarding the structure of TJS, different researchers have outlined different dimensions. Vroom [57] has classified TJS into job content, supervisor, co-worker, compensation, environment satisfaction, etc.; Locke [58] believed that TJS includes elements such as compensation, promotion, manager, benefits, recognition, co-workers, the job itself, and communication satisfaction. There was the proposed six-factor theory proposed by Bishop [59] and Chuan [60], and there was the proposed four-factor theory proposed by Easley [61].

Based on previous studies [62], this study divided TJS into two aspects of teacher education career satisfaction (TECS) and school work environment satisfaction (SWES). The former involves the experience of the teaching profession, and the latter consists of the expertise of the school in which one works.

### Teachers’ self-efficacy

The term “self-efficacy” was introduced as a social learning theory by American psychologist Bandura in 1977 in “Self-Efficacy: An Integrated Theory of Behavioral Change” and is central to Bandura’s theory of self-efficacy. Self-efficacy was explained by Bandura [63] as “the belief in an individual’s ability to organize and execute the course of action required to produce a particular achievement in a given context, and refers to the individual’s speculations and judgments about his or her ability to perform a given task.” Central to this theory is the triadic reciprocity theory, the interplay of behavioral, personal, and environmental factors, which focuses on the critical moderating role played by human cognitive processes on learning and behavior, where the person is the motivating factor of conduct and has subjective agency [64,65]. This definition applies to general self-efficacy [66] and beliefs in specific areas, such as TSE [67].

The Rand Research Group first introduced the concept of TSE in 1976. Armor et al. [68] argued that TSE refers to the extent to which teachers’ beliefs about the time to which they influence students’ academic task completion or to teachers’ beliefs about their ability to influence students well. Wolters et al. [69] saw it as the teachers’ self-judgment, thoughts, and feelings about how they accomplish their educational work and positively impact their students.

Among the many definitions of TSE, the two main influential ones are the following: The first broad category of definition focuses on the extent to which teachers trust their ability to influence student’s development: TSE was seen as a belief that teachers themselves can influence and help students and have a positive impact on student learning [70]. The second broad category assumes that TSE is a teachers’ belief about their ability to organize and perform specific teaching tasks and behaviors in a given situation. Tschannen-Moren [71] argued that this definition extends Bandura’s definition of self-efficacy and emphasizes teachers’ beliefs about their ability to teach themselves. Schwarzer et al. [72] suggested that this referred to a teachers’ perception of whether his or her teaching is eliciting successful learning and personal satisfaction from students; Troesch et al. [73] discussed TSE as teachers’ perceptions of their ability to cope with a range of challenges and difficulties in education and teaching. TALIS 2018 [74] defined TSE as the beliefs teachers hold that they can influence student’s learning outcomes (e.g., achievement, interest, and motivation) through their teaching behaviors.

In terms of characteristics, it has been demonstrated by Kleinsasser [75] that TSE is contextual, that there may be systematic differences in self-efficacy across groups, and that teachers in the same setting, such as the same school, country, or educational system, may present similar self-efficacy.

Regarding the role of TSE, research has shown [76] that it can affect teachers’ effort and persistence in teaching and influences their teaching strategies, approaches, and attitudes. School leadership (including headmasters and teachers) can directly influence TSE [77]. Teachers with a sense of efficacy are more actively engaged in educational innovation initiatives [78] and are generally comfortable with their jobs, able to manage stress reasonably well, adapt to the situation, and achieve creative teaching [79]. It significantly impacts their goal-setting, work commitment, and ability to continue teaching when they encounter difficulties [80]. High TSE is an indicator of willingness to support, implement, and create positive change, persevere through challenges, be open to new ideas, and experiment with teaching strategies even in situations that are perceived as risky [81,82].

### The Relationship between variables

#### The influence of school climate on teachers’ job satisfaction

Past research has demonstrated that TJS is strongly influenced by SC [83]. There was a significant correlation between teachers’ perceptions of SC and TSE [84]. Positive SC and support have a positive impact on TSE and motivation to teach. A supportive SC is an essential factor in improving TSE and increasing teacher motivation [85]. Teachers with a high level of collaboration with colleagues show a higher level of general teaching effectiveness than those with a low level of cooperation [86]. Teachers are more committed to their work if they feel supported by their principals and colleagues [87]. A positive SC can promote job satisfaction [88], especially when positive collegial and leadership relationships lead teachers to believe in their influence in the school, which ultimately affects their professional identity and TJS [89], while in a negative SC, it is difficult for teachers to show optimism in their educational and teaching activities [90].

Hypothesis 1-VET-perceived SC is positively predicting TJS.

#### The influence of school climate on teachers’ self-efficacy

It has been demonstrated [91] that environmental conditions affect individual self-efficacy; Ashton [92] classified the factors affecting TSE as SC, teachers’ morale, principal’s leadership style, school infrastructure conditions, and teachers’ job stress; Woolfolk et al. [93] categorized school factors affecting TSE into six areas: institutional integrity, principal influence, caring and compassion, school support system, SC, and academic emphasis. It has also been suggested that factors such as years of professional experience, challenging classroom environments, aspects of SC, and school teamwork influence TSE [94].

A study by Woolfolk et al. [95] elaborated that the structure and SC can significantly impact TSE. Hosford et al. [96] verified that SC influenced teachers’ ability and confidence in mastering the classroom. Good collaboration enhances the sense of solidarity among teachers, and teachers with positive perceptions of SC are more likely to believe in themselves and their colleagues as capable of dealing with students [97].

Hypothesis 2-VET-perceived SC is positively predicting TSE.

#### The influence of teachers’ self-efficacy on teachers’ job satisfaction

Numerous studies have demonstrated a significant relationship between TSE and TJS. When teachers perceive that their teaching is a worthwhile endeavor and that their teaching contributes to student success, the teachers themselves receive a sense of satisfaction [98]. Meristo et al. [99] revealed that teachers’ perceptions of their effectiveness in teaching, student management, and classroom management are important factors influencing their TJS. In an analysis of data from a sample of 500 teachers in Canada, Korea, and the United States, scholars have shown that collective efficacy, independent of cultural context, has a significant positive effect on TJS (which many studies have used as a proxy for well-being) [100].

Teachers with higher TSE exhibit higher TJS and organizational commitment and are less susceptible to burnout, which is critical for their well-being to be maintained and enhanced [101]. Teachers do not receive adequate support in constructing TSE, and when they experience adverse events, they gradually lose TJS and commitment [102].

According to the “perception-attitude-behavior” theory proposed by Ajzen et al. [103], human attitudes are influenced by perceptions of environmental factors, and there is a close relationship between perceptions, attitudes, and behavior. A positive SC creates a more supportive community, which makes teachers firm in their belief that they can accomplish their teaching tasks and be more satisfied with their teaching jobs [104]. That is, TSE may act as a “bridge” between teachers’ perceived SC and TJS.

Hypothesis 3-TSE is positively predicting TJS.

Hypothesis 4-TSE is significantly mediate the relationship between SC and TJS.

### Theoretical foundation and research model

**Fig 1:**
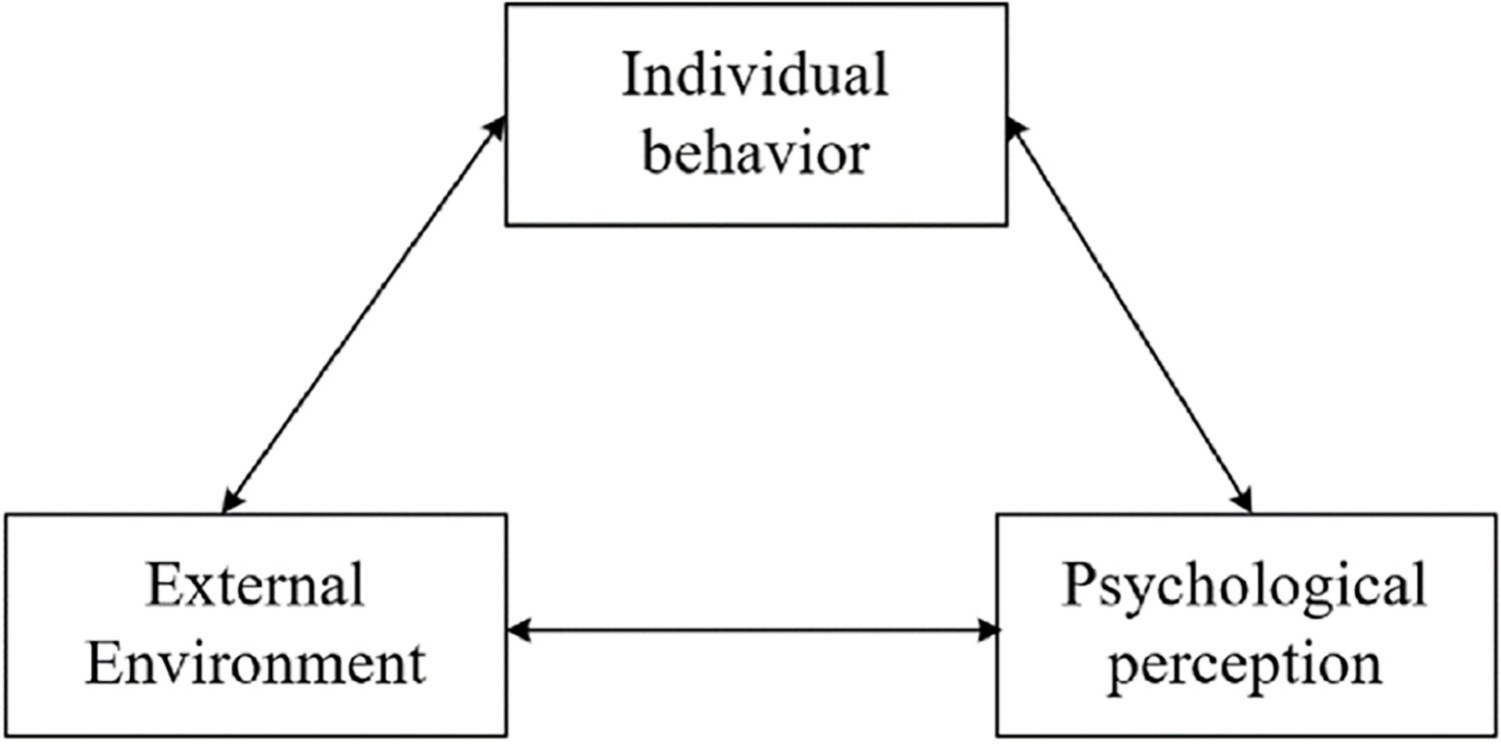
Social cognitive triadic interactive decision system.

Social Cognitive Theory (SCT) [105] was developed by Bandura in 1986, building on the earlier Social Learning Theory, which suggests that three factors—individual cognition, individual behavior, and the individuals’ external environment—interact to influence human activity, forming a triadic system of interactive decisions, as shown in Fig 1.

**Fig 2:**
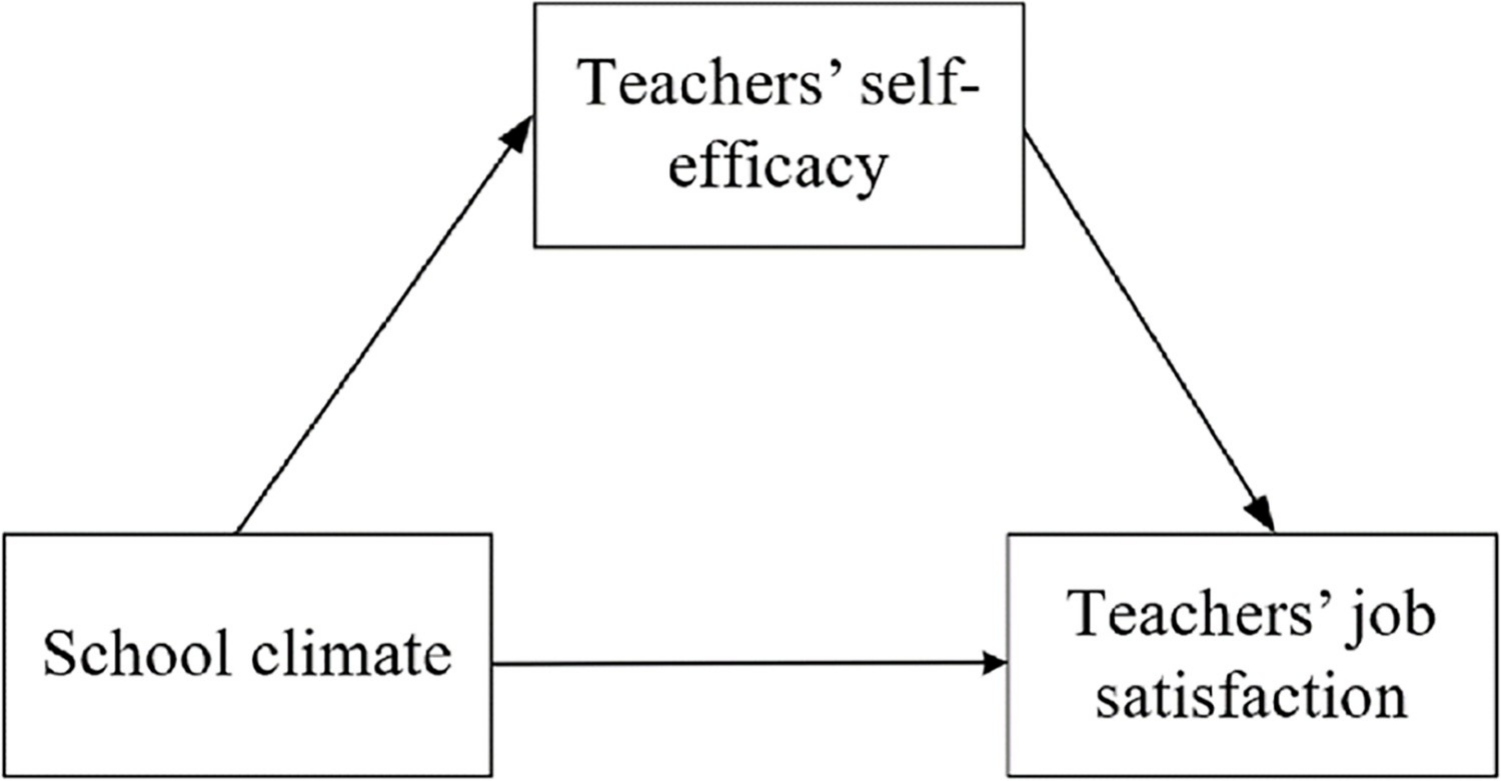
Proposed research model.

The statement of SCT on the interrelationship between the external environment, individual psychological perceptions, and individual behavior provide an excellent theoretical basis for explaining whether SC has an impact on TJS and whether SC has an effect on TSE and the underlying reasons for it in this study. Based on the above hypotheses, a hypothetical model of the effect of school climate and teacher self-efficacy on teacher job satisfaction was developed for this study is shown in Fig 2.

## Methods

### Participants and procedure

This study selected in-service VET from 12 provinces, cities, and autonomous regions in western China for analysis. In order to ensure the authenticity and validity of the findings, it was necessary to pre-test and revise the questionnaire before the formal administration. In general, the number of respondents needed to be three to five times the number of questions in the questionnaire [106]. The pre-test questionnaire was administered in March 2022 to a random sample of 100 VET in S Province, Western China. The questionnaires were processed to eliminate invalid questionnaires, resulting in 91 valid questionnaires, with an effective rate of 92.8%. The initial questionnaire was analyzed, tested for reliability and validity, and revised into an official questionnaire.

The official questionnaire survey was conducted from April 2022 to June 2022. Because this was during a period of severe COVID-19 epidemics in China, it was not practical to distribute the questionnaire in person due to national conditions and epidemic prevention policy requirements. Online questionnaires are more flexible and efficient than offline questionnaires, and the data collected is faster and more conducive to statistical analysis. To make the sample more representative, the survey adopted a combination of stratified sampling and convenience sampling for sample selection: 12 provinces (municipalities directly under the Central Government) in western China were used as a one-tier sample; provincial capital cities, prefecture-level cities, county-level cities, and townships were selected for each province (municipality directly under the Central Government) as a two-tier sample; 1–2 vocational institutions were chosen for each city/township as a three-tier sample; each school was sampled by grade level, with 2 classes as the lower limit of the number of classes per grade level, as a four-tier sample.

Following a call from the local government, review by the ethics committee, and referral by relevant experts, we first contacted the heads of the selected vocational colleges, insisting on a combination of voluntariness and anonymity, to get a rough idea of the number of teachers participating in the survey from the heads. After obtaining the teachers’ consent, the questionnaire was randomly distributed and collected via the Internet in vocational institutions in Western China. A total of 1174 questionnaires were distributed, and 1045 were collected, with a recovery rate of 89%. After taking into account the missing values, response time, and seriousness of answers, 1035 valid questionnaires were retained, with an efficiency rate of the effective rate was 99%. Among them, 357 (34.50%) were male teachers, 678 (65.50%) were female teachers, 6 (0.60%) were under 20 years old, 159 (15.40%) were 21–30 years old, 291 (28.10%) were 31–40 years old, 408 (39.40%) were 45–50 years old, 6 (0.60%) were 61 years old and above. The number of students with teaching experience of 5 years or less was 153 (14.80%), 6–10 years 134 (12.90%), 11–15 years 134 (12.90%), 31 years or more 94 (9.10%); the number of students with higher education or less was 7 (0.90%), college was 45 (4.30%), undergraduate was 843 (81.40%), and graduate was 131 (12.70%). After eliminating the invalid questionnaires, the final results were as follows. The detailed basic information of the questionnaire sample is shown in Table 1. The sample was dominated by young, medium length of service, public school, formally employed teachers, and bachelor’s degree groups.

**Table 1:** Demographics distribution of samples (N = 1035).

Demographic variables	Category	Frequency	Percentage	Demographic variables	Category	Frequency	Percentage
**Gender**	Male	357	34.50%	**Teaching experience**	< 5 years	153	14.8%
	Feminine	678	65.50%		6–10 years	134	12.9%
**Age**	<20 years	6	0.60%		11–15 years	134	12.9%
	21–30 years	159	15.40%		16–20 years	189	18.3%
	31–40 years	291	28.10%		21–25 years	195	18.8%
	41–50 years	408	39.40%		26–30 years	136	13.1%
	51–60 years	165	15.90%		>31 years	94	9.1%
**Academic qualification**	High School and below	7	0.7%	**Teachers’ title**	Unrated title	109	10.5%
	Secondary qualification	9	0.9%		Level 3 teacher	12	1.2%
	Tertiary qualification	45	4.3%		Level 2 teacher	212	20.5%
	Undergraduate qualification	843	81.4%		Level 1 teacher	416	40.2%
	Postgraduate qualification	131	12.7%		Senior Teacher	177	17.1%
**School Types**	Public School	819	79.1%		Full Senior Teacher	7	0.7%
	Private School	216	20.9%		Other	102	9.9%
**School Location**	City	431	41.6%	**Form of job appointment**	Formally on staff	737	71.2%
	County	204	19.7%		Contractual appointment	288	27.8%
	Rural (townships, towns, villages)	400	38.6%		Temporary substitute	10	1%
**School hierarchy attribute**	Key or model school	527	50.9%	**Teacher Level**	Backbone teachers at the county level and above	89	8.6%
	General School	508	49.1%		School-level backbone teachers	113	10.9%
					General teacher	833	80.5%

Note: Key schools are key because they enjoy policy benefits and financial support that many schools do not. These schools have good student populations, high promotion rates, good school culture, and more government attention.

### Ethics statement

The objectives and procedures of this study were reviewed and approved by the Xi’an Education Supervisory Board Review Committee prior to the investigation of this study. All questionnaires were distributed anonymously via the Internet to each subject, who provided confidential information on a voluntary basis after understanding the purpose of the survey and related procedures, and had the right to terminate the process at any time during the survey.

### Measures

Based on the international survey report and related literature or scales, the “Professional Development Questionnaire for VET” is compiled, including SC, TJS, and TSE. More specific psychometric information is shown in Table 2. The total scale demonstrated high reliability (Cronbach alpha coefficient is 0.951), indicating that the reliability is ideal. According to the degree of conformity with the actual feelings of the VET, all items used a 5-point Likert-type scale from Totally disagree (1) to agree Strongly(5). The higher the score, the higher the degree of conformity with the VET’s feelings. In order to avoid the influence of the subjects’ thinking when answering the questions, some of the items in the questionnaire were narrated in reverse, and the corresponding score conversion was made in the scoring. The details are as follows:

**Table 2:** Total questionnaire specific psychometric information.

Questionnaire	Dimensions	Title items	Number of items
Teacher Background		Gender、 Age 、Teaching experience、academic qualification、Teachers’ title 、School Location 、School hierarchy attribute 、School Types 、Form of job appointment、Teacher Level 、Monthly Salary	11
School Climate	Interpersonal relationship climate	CR1: I get along well with my colleagues and have good communication	6
		CR2: My colleagues and I trust each other and help each other out	
		TSR: I get along well with my students	
	Decision-making sharing climate	DMSC1: School leaders respect the views of teachers when making decisions about their work	
		DMSC2: School leaders can consider my dignity	
		DMSC3: School leaders can care about the living conditions of staff	
Teachers’ Self-Efficacy		TSE1: I am able to infuse classroom teaching with international understanding concepts	4
		TSE2: I can guide and help students to set individualized learning plans	
		TSE3: I am proficient in the use of computers, whiteboards, projection and other equipment	
		TSE4: I can assign graded, flexible and personalized work	
Teachers’ Job Satisfaction	Teacher education career satisfaction	TECS1: I am actively supported by the school in my self-development and promotion	5
		TECS2: I am satisfied with the teaching and research mechanism in my school	
		TECS3: I am satisfied that my school is able to assess and recognize junior titles on its own	
	School work environment satisfaction	SWES1: In the past three years, the school has built a standardized sports hall and library	
		SWES2: In the last three years, the school has been well-equipped with IT equipment	

Note: CR = colleague relationship, TSR = teacher-student relationship.

#### School climate

The scale for this study was adapted from the literature of Johnson et al. [40] based on the characteristics of SC and the actual situation of VET in western China regarding existing established scales. Factor analysis was first performed, and items were retained if they presented high loadings on only one factor and low loadings on the others and if they did not reduce the internal consistency coefficient of the scale. Next, an exploratory factor analysis (EFA) was conducted, and a two-factor solution was selected as the best solution, with those items from the original colleague relationship (CR) and teacher-student relationship (TSR) coming together to form a common factor, both reflecting the dimension of interpersonal relationship climate (IRC). The scale consisted of two dimensions: IRC and decision-making sharing climate (DMSC), where DMSC refers to the extent to which teachers participate in school decision-making, reflecting the formation of democratic decision-making in the school. Then verify its adaptability among vocational education teachers in western China.

The Cronbach’s alpha reliability coefficient of the scale in the present study is 0.913, and the KMO value is 0.836. It is carried out utilizing teacher self-assessment. According to the suggestion of Fornell et al. [107], The AVE (average variance extracted) and the CR (composite reliability) were used to test the reliability of the SC scale and each dimension, and the results are shown in Table 3, which shows that the scale has high reliability. The factor loads for the two dimensions are shown in Table 3 between 0.820–0.970 and 0.934–0.946, respectively. The cumulative contribution rate is 72.003%, and it is significant at the level of 1%. By rotating the factor load matrix by principal component analysis and maximum variance method, the results show that the structural validity of this scale is good; Table 4 shows that the various structural fitting indices are promising, indicating that the reliability and validity of the scale are good. The results demonstrate that the scale applies to vocational education teachers in western China.

**Table 3:** Assessment of measurement model.

Scale	Dimension	Item	Estimate	AVE	CR
**SC**	IRC	CR1	0.955	0.8418	0.9407
		CR2	0.97		
		TSR	0.82		
	DMSC	DMSC1	0.934	0.8824	0.9575
		DMSC2	0.938		
		DMSC3	0.946		
**TSE**		TSE1	0.708	0.5865	0.8494
		TSE2	0.774		
		TSE3	0.715		
		TSE4	0.857		
**TJS**	TECS	TECS1	0.896	0.7518	0.9005
		TECS2	0.912		
		TECS3	0.788		
	SWES	SWES1	0.87	0.8393	0.9124
		SWES2	0.96		

Note: SC = school climate, TSE = teachers’ self-efficacy, TJS = teachers’ job satisfaction, IRC = interpersonal relationship climate, DMSC = decision-making sharing climate, TECS = teacher education career satisfaction, SWES = school work environment satisfaction, CR = colleague relationship, TSR = teacher-student relationship.

**Table 4:** Confirmatory factor analysis (CFA) results (N = 1035).

Scale	Fit Indices							
	X^2^/df	GFI	AGFI	NFI	IFI	TLI	CFI	RMSEA
**SC**	5.063	0.987	0.967	0.994	0.995	0.991	0.995	0.063
**TSE**	2.348	0.998	0.989	0.997	0.998	0.995	0.998	0.036
**TJS**	5.847	0.991	0.966	0.994	0.995	0.988	0.995	0.068

Note: SC = school climate; TSE = teachers’ self-efficacy; TJS = teachers’ job satisfaction; GFI = goodness of fit index; AGFI = adjusted goodness of fit index; NFI = Normed Fit Index; IFI = incremental fit index; TLI = Tucker Lewis Index; CFI = Comparative fit index; RMSEA = Root mean square error of approximation.

It is also worth noting that focusing only on the collective nature of SC would miss much important information [108]. Each dimension of SC represents one of the attributes of the school; therefore, referring to the relevant literature [109], an ANOVA was used to confirm that these attributes represented a collective school rather than an individual teacher phenomenon. The analysis of IRC results is shown in Table 5, where the variation between schools is much more significant than the variation within schools. 352.841 is a statistically significant rate that exceeds the 0.001 level. Similarly, the DMSC analysis result is shown in Table 6, where the ratio of 948.014 is statistically significant and exceeds the 0.001 level, and the expected assumptions are confirmed.

**Table 5:** ANOVA on IRC.

	Sum of square numbers	D.F.	Average square	F Ratio	Significance
Inter group	364.701	7	52.1	352.841	0.000
Within Group	151.646	1027	0.148		
Total	516.347	1034			

Note: IRC = interpersonal relationship climate.

**Table 6:** ANOVA on DMSC.

	Sum of square numbers	D.F.	Average square	F Ratio	Significance
Inter group	473.784	12	39.482	948.014	0.000
Within Group	42.563	1022	0.042		
Total	516.347	1034			

Note: DMSC = decision-making sharing climate.

#### Teachers’ self-efficacy

Although different scholars have different opinions on the definition of TSE, the mature international scale primarily uses unidimensional divisions for measurement [110]. An EFA of all items also revealed a one-factor structure. In addition, considering the conceptual clarity of the items and taking into account the characteristics of VET in western China, the items with low internal consistency coefficients were censored. The scale mainly examines TSE in classroom management, teaching and student management, and homework.

The alpha coefficient of 0. 846 and a KMO value of 0.818. The results of AVE and CR are shown in Table 3, which shows that the questionnaire has high reliability. The cumulative contribution rate is 68.972%, and it is significant at the level of 1%. By rotating the factor load matrix by principal component analysis and maximum variance method, the results show that the structural validity of this scale is also good. The confirmation factor load is between 0.708–0.857, and the structural fitting indices show promising results, as shown in Table 4, indicating that the reliability and validity of the scale are good.

#### Teachers’ job satisfaction

Referring to the international TALIS [111] survey report development scale, which has five items with Cronbach’s alpha coefficient of 0.920 and a KMO value of 0.844; it is self-reported by teachers and consists of two main dimensions: teacher education career satisfaction (TECS) and school work environment satisfaction (SWES).

The results of the AVE (average variance extracted) and the CR (composite reliability) test for TJS and the reliability of each dimension are shown in Table 3, indicating that the scale has high reliability. The cumulative contribution is 76.469% and is significant at the 1% level. The factor loading matrix rotation results by principal component analysis and maximum variance method showed that the scale also had good structural validity. The factor loadings of the two dimensions ranged from 0.788–0. 912 and 0.870–0.960, respectively, and the structure fit indices are good, as shown in Table 4, indicating good reliability of the scale.

### Data processing

Using SPSS 26.0, Amos 24.0, and other software for data management and analysis, the primary analysis methods include reliability analysis, confirmative factor analysis (CFA), descriptive statistics, correlation analysis, and structural equation model (SEM), and Bootstrap test (mediation effect test).

### Common method bias test

In this study, the subjects self-reported scale, which may lead to common method bias in the predictor and outcome variables due to the same reporting source, thus reducing the study’s validity. Therefore, a common method bias test should be performed on the sample data before data analysis. This study mainly adopts the Harman [112] one-factor test method. This method assumes that a common method bias exists if a factor analysis of all variables of the scale results in the study of only one factor or if the explanatory power of one factor is exceptionally high.

In this study, firstly, exploratory factor analysis is performed to test the data with KMO and Bartletts’ spherical test, and the results showed that KMO = 0. 940, Bartletts’ value is 15254.490, df = 105, p < 0. 001, so the data are suitable for factor analysis. The analysis revealed three common factors with eigenvalues greater than 1, of which the first common factor explained 36.223% of the variance, which is less than the determined 50% judgment criterion [113]. Secondly, the results of confirmative factor analysis (CFA) showed that the fit indicators of the one-factor model are poor (x 2 = 4460.104, x 2/df = 49.557, GFI = 0.587, AGFI = 0.450, CFI = 0.713, TLI = 0.665, RMSEA = 0.217, SRMR = 0.096). Therefore, there is no apparent common method bias among the study variables.

## Results

### Preliminary analysis

#### Descriptive statistics and correlation analysis

The means, standard deviations, and correlations among variables for SC, TSE, and TJS are shown in Table 7. The data results showed that the highest mean value of the DMSC dimension (M = 6.237, SD = 1.464) and the lowest mean value of the IRC dimension (M = 4.559, SD = 0.597) were found for teachers’ perceived SC. Among TJS, TECS (M = 4.202, SD = 0.843) is higher than SWES (M = 4.063, SD = 1.018). The Pearson product difference correlation results showed that the correlations between SC, TSE, and TJS were significant (p < 0. 01) and positively correlated. According to Cohen [114], the magnitude of the product difference correlation coefficient itself reflects the size of the “effect size” and can be directly used as an effect size. The effect sizes of the correlation coefficients in this study were all above medium according to Cohen (ρ = 0. 1 for small; ρ = 0. 3 for medium; and ρ = 0. 5 for large), and the statistical power of all correlations was above 0. 99. These correlations are consistent with the H1, H2, and H3 of this study, which also provided the necessary preconditions for the subsequent development of the structural equation model.

**Table 7:** Means, standard deviations and correlation coefficients of the main variables (N = 1035).

	1	2	3	4	5	6	7	8	9	10	11	12	13	14	15	16	17
1	1																
2	0.952**	1															
3	0.924**	0.762**	1														
4	0.701**	0.709**	0.597**	1													
5	0.826**	0.811**	0.734**	0.654**	1												
6	0.595**	0.609**	0.497**	0.630**	0.830**	1											
7	0.833**	0.801**	0.759**	0.562**	0.940**	0.590**	1										
8	-0.017	-0.022	-0.008	-0.037	-0.056	-0.065*	-0.042	1									
9	-0.110**	-0.082**	-0.129**	-0.053	-0.035	0.069*	-0.093**	-0.235**	1								
10	-0.102**	-0.069*	-0.129**	-0.033	-0.019	0.096**	-0.086**	-0.206**	0.903**	1							
11	-0.019	-0.032	-0.002	-0.044	-0.037	-0.047	-0.025	0.076*	-0.121**	-0.175**	1						
12	0.017	0.027	0.002	-0.039	0.037	0.055	0.021	-0.027	0.245**	0.255**	0.092**	1					
13	-0.082**	-0.082**	-0.070*	-0.095**	-0.028	0.007	-0.045	-0.090**	0.088**	0.106**	-0.092**	0.106**	1				
14	0.090**	0.061*	0.113**	0.005	0.029	-0.085**	0.094**	0.058	-0.370**	-0.434**	0.140**	0.115**	-0.216**	1			
15	0.136**	0.134**	0.120**	0.125**	0.097**	0.077*	0.094**	0.013	0.03	0.041	-0.106**	-0.156**	0.249**	-0.190**	1		
16	0.123**	0.093**	0.143**	0.046	0.048	-0.067*	0.110**	0.065*	-0.415**	-0.467**	-0.024	-0.023	-0.209**	0.755**	-0.069*	1	
17	-0.002	0.005	-0.01	-0.008	0.003	-0.008	0.009	0.069*	-0.103**	-0.110**	-0.045	-0.119**	-0.017	-0.048	0.047	0.002	1
M	4.1463	4.2019	4.0628	4.3251	4.3585	4.5591	6.2367	1.6551	3.5652	3.8918	4.0454	3.9362	1.97	1.2087	1.4908	1.2976	2.7188
SD	0.85772	0.84299	1.01793	0.65729	0.70666	0.59749	1.46395	0.47557	0.97073	1.87106	0.51371	1.51069	0.89598	0.40657	0.50016	0.47809	0.61192

Note:1. Teachers’ Job Satisfaction 2. Teacher education career satisfaction 3. School work environment satisfaction 4. Teachers’ Self-Efficacy 5. School Climate 6. Interpersonal relationship climate 7. Decision-making sharing climate 8. Gender 9. Age 10. Teaching experience 11. Academic qualification 12. Teachers’ title 13. School Location 14. School hierarchy attribute 15. School Types 16. Form of job appointment 17. Teacher Level; M = Arithmetic mean, SD = Standard deviation

* p<0.05

** p<0.01

***p<0.001 (two-tailed test).

#### Structural equation modeling analysis

**Fig 3:**
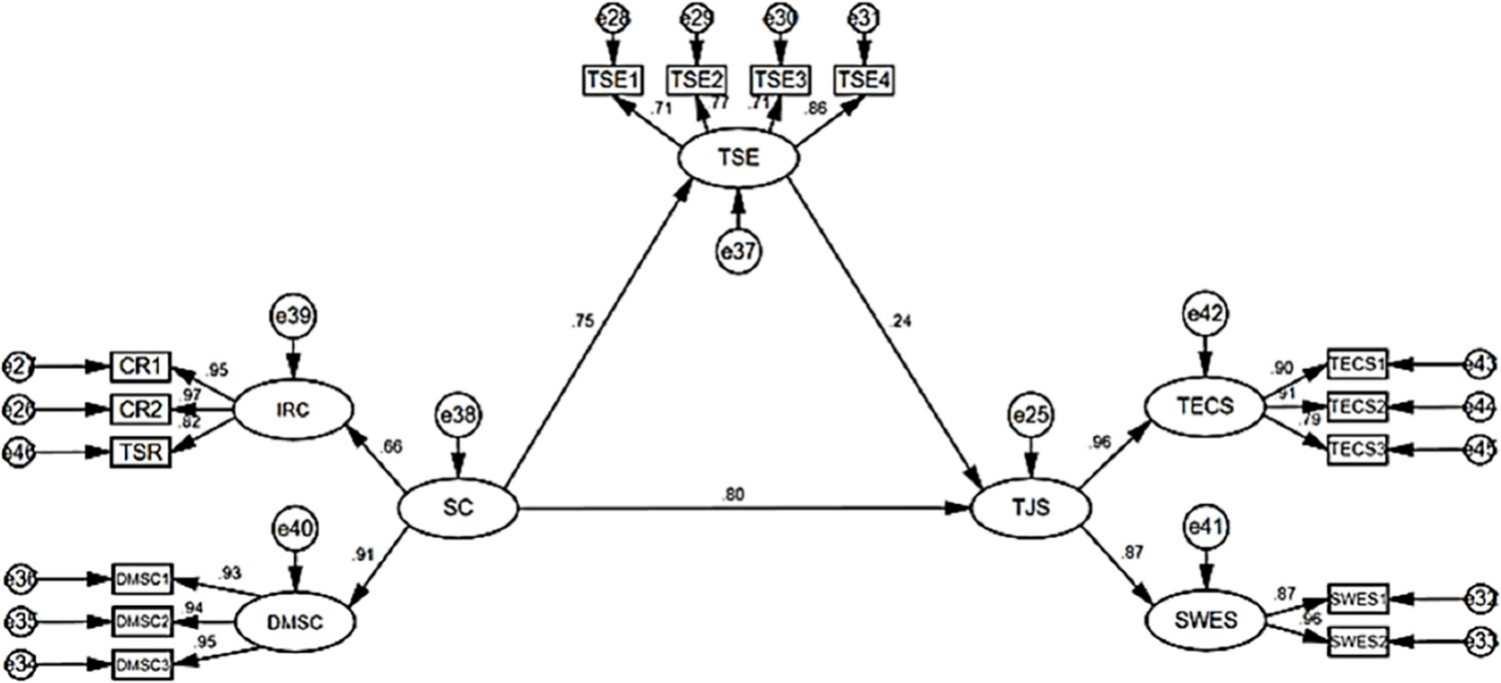
Mediation model path of Teacher’s self-efficacy.

Using Amos 24.0 to test the structural equation model, the ratio of chi-square degrees of freedom is 509.758, and the P value is 0.000. Wu, Ming-Lung [115], Hox, et al. [116], and Schreiber [117] point out that in the case of large samples (N ≥ 1 000), the ratio of cardinal degrees of freedom is only used as a reference indicator and is usually not used to prove the fit of the data to the model, and that P < 0. 001 is standard. Referring to the research of Wen Zhonglin et al. [118], the NFI, CFI, GFI, and TLI of the model with a better fitting degree should be greater than 0.9. The structural equation model tests the relationship between SC, TSE, and TJS perceived by VET. The statistical results are shown in Table 8, and the fitting degree index of the structural equation model is good. The normalized path coefficients between variables are shown in Fig 3.

**Table 8:** Structural equation model fitting indices.

Indices	p	CFI	GFI	NFI	TLI	IFI	RMSEA	SRMR
**Fitting value**	0.000	0.972	0.941	0.967	0.965	0.972	0.071	0.059
**Range of indices**	<0.05	>0.9	>0.9	>0.9	>0.9	>0.9	<0.08	<0.08

Note: CFI = Comparative fit index; GFI = goodness-of-fit index; NFI = Normed Fit Index; TLI = Tucker Lewis Index; IFI = incremental fit index; RMSEA = Root mean square error of approximation; SRMR = Standardized root mean residual.

**Fig 4:**
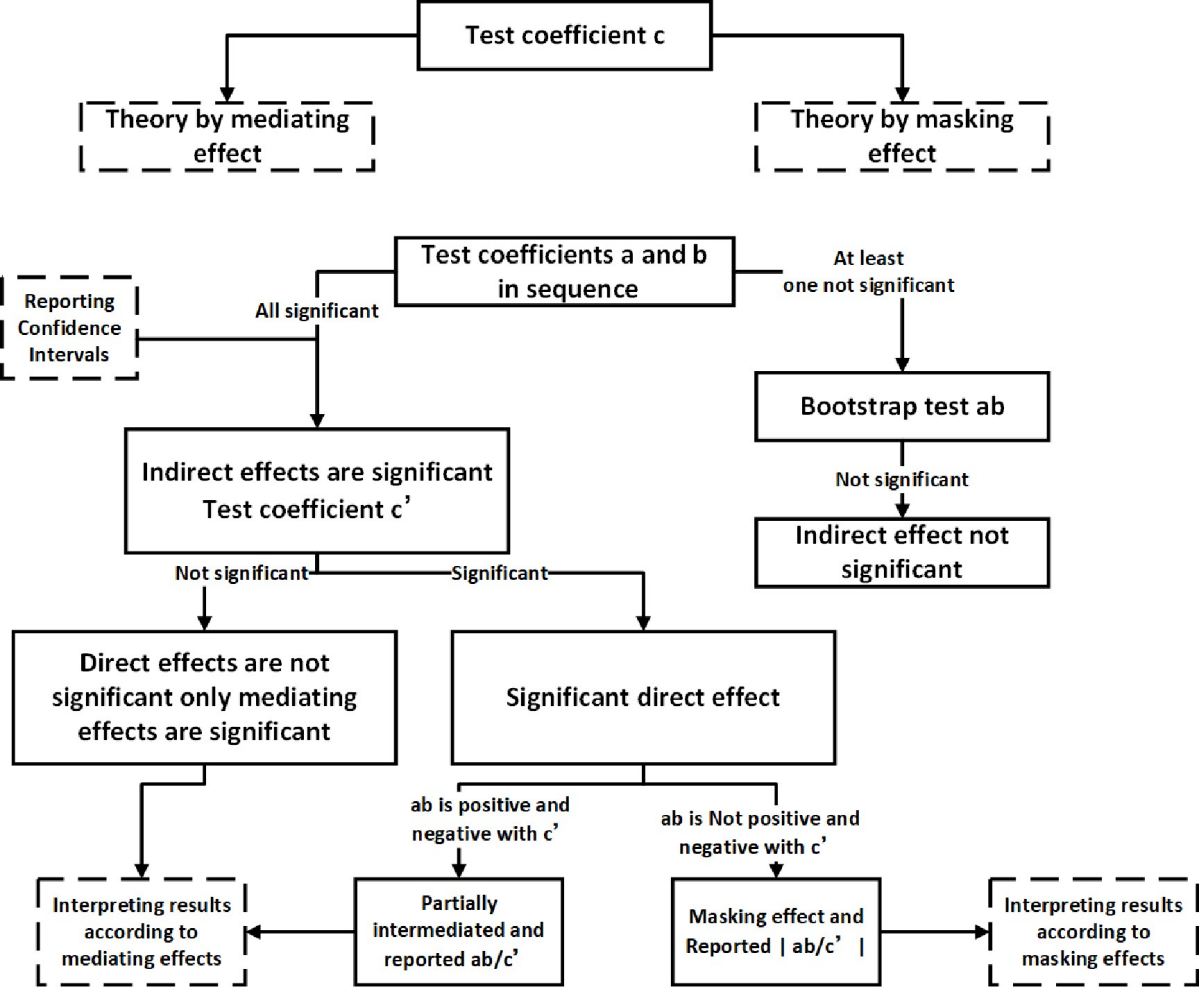
Mediation effect test process (an improvement on Baron and Kenny’s causal steps approach).

The causal steps approach proposed by Baron et al. [119] in 1986 is one of the most popular methods for testing mediation effects, but scholars [120–122] have gradually questioned it. Some scholars believe that its testing power is the lowest among various methods, i.e., it is less easy to test for mediating effects using this method. Another drawback is that Baron et al. consider the significance of c as a prerequisite for testing mediating effects. At the same time, the study by MacKinnon et al. [123] confirms that this prerequisite is unnecessary, and there may be suppressing effects. To overcome these shortcomings, this study uses a modified causal steps approach proposed by scholars [124] in 2014, which considers that the significance of the total effect c is not a prerequisite for the existence of mediating effects, as shown in Fig 4. The results are shown in Table 9: the coefficient c of the equation in the first step is 0.986, significant at the 95% confidence interval (0.945, 1.028), and is established as a mediating effect. In the second step, the coefficients a and b of the equation are examined in turn. A coefficient a of 0.6 and significant at the 95% confidence interval (0.556, 0.643) and a coefficient b of 0.775 and effective at the 95% confidence interval (0.725, 0.826) indicate a significant indirect effect. The third step tests that the equation c coefficient is 0.352 and powerful at the 95% confidence interval (0.297, 0.406), indicating a significant direct effect. The fourth step tests the sign of ab and c’, ab = 0.465 and c’ = 0.352 with the same sign, a partial mediating effect, and the ratio of the mediating effect to the total effect is 0.47 (ab/c). In summary, hypotheses 3 and 4 were supported.

**Table 9:** The mediating effect of TSE between SC and TJS(N = 1035).

variables	TSE			TJS			
	Model1a		Model1b	Model2a		Model2b	Model2c
Control variables							
**Gender**		-0.084	-0.022	-0.092	0.010		0.017
**Age**		-0.095	-0.040	-0.104	-0.014		0.000
**Teaching experience**		0.013	0.015	-0.028	-0.024		-0.030
**Highest Degree**		-0.053	-0.017	-0.060	0.000		0.005
**Title**		0.001	-0.012	0.035	0.014		0.018
**School Location**		-0.097***	-0.073***	-0.103**	-0.063***		-0.037*
**School Attributes**		-0.060	-0.060	0.005	0.006		0.027
**School hierarchy Attributes**		0.196***	0.102**	0.299***	0.144***		0.108***
**Form of job appointment**		0.043	0.027	0.104	0.076		0.067
**Level**		-0.013	-0.027	0.002	-0.021		-0.012
**Average monthly income**		0.082	-0.018	0.161***	-0.004		0.003
**Independent variable**							
**SC** **(95% confidence interval)**			0.6***		0.986***		0.775***
			(a)		(c)		(b)
			(0.556, 0.643)		(0.945, 1.028)		(0.725, 0.826)
**mediator variable**							
**TSE** **(95% confidence interval)**							0.352*** (c’)
							(0.297, 0.406)
**R^2^**		0.048	0.445	0.071	0.701		0.741
**ΔR^2^**		0.048	0.397	0.071	0.630		0.040
**ΔF**		4.680	731.116	7.083	2156.136		159.082

Note

*p<0.05

**p<0.01

***p<0.001

The coefficients in the table are standardized regression coefficients.

The Bootstrap procedure [125] is used to test the mediating effects of TSE. First, 5000 Bootstrap samples are drawn from the original data (N = 1035) using repeated random sampling, and then the model is fitted to these samples to generate and save 5000 estimates of the mediation effects, forming an approximate sampling distribution. The 95% confidence interval for the mediated effect is estimated using the 2nd and 97.5th percentiles. Suppose the confidence interval in the result does not contain 0. In that case, the product of the coefficients is significant [126], called the non-parametric percentile Bootstrap method, which has higher test power than the Sobel test [127]. The higher test power is the confidence interval using bias correction, called the bias-corrected non-parametric percentile Bootstrap method [128]. As can be seen from Table 10, the confidence interval does not contain 0, and the partial mediation effect is significant, regardless of whether the non-parametric percentile Bootstrap method or the bias-corrected non-parametric percentile Bootstrap method is used. In summary, combining the modified causal steps approach with the Bootstrap method can confirm that the mediation effect is significant and partially mediated.

**Table 10:** Bootstrap analysis of direct, indirect, and total effects.

SC-TJS	Point estimate	Product of coefficient multiplication		Bootstrapping				
				percentile 95% CI		Bais-corrected percentile 95% CI		Two-tailed significance
		SE	Z	Lower	Upper	Lower	Upper	
**Total effect**	1.822	0.101	18.040	1.615	2.079	1.607	2.068	0.001
**Direct effect**	1.488	0.165	9.018	1.202	1.867	1.177	1.812	0.001
**mediating effect**	0.334	0.117	2.855	0.099	0.503	0.117	0.513	0.008

Note: Bootstrapping = 5000; *p <0. 05, **p <0. 01, ***p<0. 001.

## Discussion and conclusion

### Discussion of key findings

This study first analyzed the basic situation of TJS in western China, then examined the relationship between teachers’ perceived SC, TJS, and the role played by TSE.

#### Characteristics of vocational education TSE in western China

This study analyzed the basic TJS of VET in western China in terms of gender, age, academic qualification, teaching experience, school types, and location. The results showed that age, school location, teaching experience, school type, school hierarchy attribute, and form of job appointment significantly affected TJS. The TJS between the ages of 51 and 60 is lower than that of teachers in other age groups, and the TJS with more than 30 years of teaching experience is lower than that of teachers with different teaching experiences. The reason for this may be that as teachers grow older and more familiar with their environment and daily work, they may quickly lose their sense of novelty, become tired and bored with the affairs around them, and develop negative emotions such as burnout, which in turn reduces their job satisfaction. The TJS located in city areas is higher than that of teachers in rural areas, probably because there is a gap between rural teachers and city teachers regarding the working environment and job treatment. Thus a stronger sense of dissatisfaction is generated. In addition, the correlation analysis also proved that the school type, form of job appointment, and hierarchy attribute were significantly and positively associated with TJS. The reason for this may be that teachers in public schools and those who accept formal appointments are backed by government finances, have high stability, have more explicit income expectations, and therefore have relatively high job satisfaction. In contrast, teachers in private schools and temporary appointees are subject to tremendous psychological stress because their salary sources are closely related to the schools’ operating conditions. The instability of pay can significantly reduce teachers’ job satisfaction ratings.

#### The relationship between SC and TJS and the mediating role of TSE

The results of this study showed that SC is significantly correlated with TJS. Both dimensions of SC (IRC, DMSC) are significantly correlated with both dimensions of TJS (TECS, SWES). This result is consistent with the results of previous studies on SC and TJS [129–131], indicating that SC does have a close relationship with TJS. It is an essential situational factor for increasing TJS.

SC in this study included DMSC and IRC dimensions, which are significantly and positively related to TJS. The leadership style of school principals deeply influences the dimension of DMSC. As the soul of the school, the principal’s leadership behavior can shape the SC. Principals caring about leadership is positively related to an open SC [132], and teachers who work in available schools have high job satisfaction [133]. Solidified management ideology, a lagging school management model, and a strict “hierarchy” make it difficult for young teachers to develop a sense of belonging due to a lack of autonomy [134], which in turn can reduce TJS.

Therefore, We should make efforts to create a democratic and harmonious SC. Due to the geographical constraints in western China, improving TJS relies on external forces while striving to achieve endogenous TJS within the school by creating a positive SC. As the most immediate environment in which teachers live, the SC plays a vital role in unifying, integrating, assimilating, and regulating the behavior and psychology of the school community. The higher the degree of democracy, cooperation, and sharing in the school, the better the SC teachers perceive, and the more they tend to cooperate and communicate with others in the work process, the more positive experiences they gain, the higher their enthusiasm for work and the higher TJS.

In addition, the results of this study indicated that the perceived SC of VET in western China had an indirect positive effect on TJS by influencing their TSE, which confirmed the research hypothesis. TJS in this study includes two dimensions: teacher education career satisfaction (TECS) and school work environment satisfaction (SWES). The correlation analysis reveals that the correlation between TECS and TSE is more significant than that between SWES and TSE, which may be since TSE, as an individuals’ personal and active will, acts more directly and comprehensively on individuals than school-level work environment factors and thus is more likely to affect individual TJS. In a well-integrated school environment, teachers are more likely to take the initiative to develop and demonstrate their personal competencies and to strive for support to achieve their goals. Teachers who are not supported in their teaching feel unmotivated [135,136]. When teachers are satisfied with the economic benefits of their work and the interpersonal relationships of their colleagues, their morale is bound to increase [137]. In this positive SC, teachers can take full advantage of their own motivation, proactively regulate their mindset, and increase their TSE.

Therefore, regional differences and the unique characteristics of vocational education teachers should be fully considered. Actively advocate and help VET in economically backward areas to create an SC of cooperation, sharing and harmony, enhance the TSE of VET, and make them believe that they can carry out effective education and teaching, student management and class management, and then feel the self-confidence brought by successful teaching and administration. In addition, this research has found that TJS can be influenced by multiple factors simultaneously. It is inappropriate to focus only on the role of individual elements; we should consider the combined effect of various factors. The correlation analysis shows that the TJS is significantly related to personal development and welfare, so it is necessary to increase the investment in education funds in the economically backward region of western China, encourage schools to conduct self-research projects, improve the ways of income generation, optimize the salary distribution system of teachers, eliminate the phenomenon of “unfair” distribution of job allowances, enhance TSE and achieve the goal of improving their satisfaction.

### Theoretical implications

This study will help to enrich the theoretical system of factors influencing TJS of VET. The combination of objective and individual subjective factors is of great value in analyzing TJS in a multidimensional and in-depth manner. The study examines the influence of SC and TSE on TJS from the perspective of individual VET. The study also investigates whether TSE can influence the effect of SC on TJS. These have important theoretical implications for the study of corresponding theoretical systems.

### Practical implications

Firstly, for governments at all levels, based on the data and findings of this study, relevant departments can have a more accurate understanding of the current situation of TJS in vocational education, as well as the intrinsic influence of SC and TSE, which provide specific empirical data to support the formulation of management measures by relevant departments and have particular reference and value for the formulation and implementation of relevant policies by governments at all levels.

Secondly, for school administrators, the analysis and findings of this study will enable them to recognize the current state of TJS and the main influencing factors so that they can ameliorate their management decisions, implement appropriate measures to meliorate SC, enhance TSE, uplift overall TJS and consequently strengthen operational efficiency.

### Limitations and future research

It is important to note that this study primarily used a questionnaire approach, and future consideration needs to be given to incorporating qualitative research methods to deepen understanding. Differences in SC, TSE, and TJS among teachers of different subjects and other variables need to be further explored.

## Conclusion

This study focuses on a specific group of VET in an economically backward region of western China. TJS of VET in Western China is moderate and influenced by a number of factors. SC was significantly related to TJS and TSE. TSE partially mediated the relationship between SC and TJS.

## Supporting information

S1 Dataset (XLSX)
